# Functional impact of multi-omic interactions in breast cancer subtypes

**DOI:** 10.3389/fgene.2022.1078609

**Published:** 2023-01-05

**Authors:** Soledad Ochoa, Enrique Hernández-Lemus

**Affiliations:** ^1^ Computational Genomics Division, National Institute of Genomic Medicine, Mexico City, Mexico; ^2^ Programa de Doctorado en Ciencias Biomédicas, Universidad Nacional Autónoma de México, Mexico City, Mexico; ^3^ Center for Complexity Sciences, Universidad Nacional Autónoma de México, Mexico City, Mexico

**Keywords:** multi-omics, breast cancer, network biology, HIF, RAS, WNT, SOX9, DNA methylation

## Abstract

Multi-omic approaches are expected to deliver a broader molecular view of cancer. However, the promised mechanistic explanations have not quite settled yet. Here, we propose a theoretical and computational analysis framework to semi-automatically produce network models of the regulatory constraints influencing a biological function. This way, we identified functions significantly enriched on the analyzed omics and described associated features, for each of the four breast cancer molecular subtypes. For instance, we identified functions sustaining over-representation of invasion-related processes in the basal subtype and DNA modification processes in the normal tissue. We found limited overlap on the omics-associated functions between subtypes; however, a startling feature intersection within subtype functions also emerged. The examples presented highlight new, potentially regulatory features, with sound biological reasons to expect a connection with the functions. Multi-omic regulatory networks thus constitute reliable models of the way omics are connected, demonstrating a capability for systematic generation of mechanistic hypothesis.

## 1 Introduction

The establishment of high-throughput technologies has made possible a systems biology approach to cancer through multi-omics integration (Kristensen et al., 2014). The multi-omics perspective takes advantage of the complementarity between different molecular levels of description. However, the promise of attaining mechanistic explanations (Bersanelli et al., 2016) has not settled yet.

Although there is a plethora of statistical approximations (Huang et al., 2017), sparse multivariate methods are arguably nearer to the mechanistic explanation goal, given their capacity to pinpoint potential regulators (Li et al., 2012; Sohn et al., 2013; Bose et al., 2022). These approaches have even identified potential key regulators for each breast cancer subtype (Huang et al., 2019), and for the subgroups of the triple-negative breast cancer subtype (Chappell et al., 2021). The networks shown in some of these works (Li et al., 2012; Sohn et al., 2013; Huang et al., 2019) constitute hypothesized models of the way regulators are connected, demonstrating a capability for systematic production of testable regulatory mechanisms.

**FIGURE 1:**
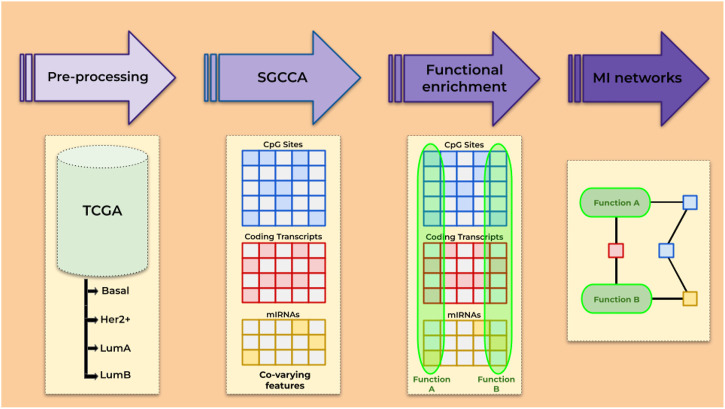
Overview of the steps followed.

Here, we applied the sparse generalized canonical correlation analysis (SGCCA) to data on DNA methylation and gene and miRNA expression from TCGA. The SGCCA is a statistical method that outputs correlated features among a large collection by the use of LASSO penalization (Tenenhaus et al., 2014). The SGCCA has been successfully used for biomarker discovery from cancer (Fan et al., 2020) and non-cancer contexts (Garali et al., 2018). In order to find not just the features but the connections between them, SGCCA was coupled with ARACNE (Margolin et al., 2006), a method for inference of transcriptional networks, that has allowed our group to find transcriptional master regulators (Tapia-Carrillo et al., 2019), to document a loss of long-distance co-expression (García-Cortés et al., 2020; Dorantes-Gilardi et al., 2021; García-Cortés et al., 2021), and to evaluate the role that relevant miRNAs play in some oncogenic processes (Drago-García et al., 2017; Zamora-Fuentes et al., 2022), among other applications in the large-scale molecular study of cancer. As an outcome, we describe some of the reconstructed networks and their implications, highlighting their relevance to understand cancer biology and potentially impact treatment. The general pipeline is described in Figure 1.

## 2 Methods

All the analyses described hereafter were performed with R programming language version 4.1.1 (R Core Team, 2021) and can be found at http://csbig.inmegen.gob.mx/SGCCA/. Release 105 of biomaRt was used all along and plots were produced with ggplot2 (Wickham, 2016).

### 2.1 Data acquisition

TCGA data were obtained through the TCGAbiolinks R package. We only used samples with Illumina Human Methylation 450, RNA-seq, and miRNA-seq data from unique patients. This constraints the number of samples to 128 from the basal subtype, 46 from Her2-enriched, 416 from luminal A, 140 from luminal B, and 75 samples from normal adjacent tissue.

Pre-processing has been described before (Ochoa et al., 2021) and follows published guidelines (Aryee et al., 2014; Tam et al., 2015; Tarazona et al., 2015). As a first step, only protein-coding transcripts were kept since for our purposes, these were considered the main functional effectors. This restriction toward the study of non-coding features was chosen in order to focus on the expression regulatory layers of DNA methylation, miRNA expression and, hidden among the transcripts, the layer of transcription factors. Length and GC content biases were checked with the NOISeq package (Tarazona et al., 2015) and alleviated using EDASeq (Risso et al., 2011) full normalization. Genes with zero counts were (the only ones) discarded at the low count filter, TMM normalization was applied between samples, and the batch effect was corrected. Since batch effects can be induced by *a priori*-unknown factors, ARSyNseq was used to remove all systematic noise not associated with the subtypes (Nueda et al., 2012). Preprocessing of microRNAs is the same, except there is no length or GC bias and the normalization used between samples is the median method.

Finally, CpG probes with over 25% missing values and non-mapped or overlapping SNPs were discarded. The remaining missing values were imputed *via* nearest neighbors and transformed into M-value matrices. This way, datasets account for 393,132 methylation probes, 17,077 coding transcripts, and 604 miRNA precursors.

### 2.2 Sparse generalized canonical correlation analysis

Once pre-processing was performed, we normalized each omic by the square root of the first eigenvalue and concatenated them patient-wise, obtaining one matrix per breast cancer subtype and one for the normal tissue. Using this normalization ensures the influence of each omic over upcoming analysis depends on its variance (De Tayrac et al., 2009).

Afterward, we approached the SGCCA as implemented in the mixOmics package (Rohart et al., 2017) and largely followed the Garali et al. guidance (Garali et al., 2018). The analysis takes as input the different blocks of data and a sparsity parameter per block, the number of components to recover (ncomp), a design matrix, and a function to maximize the covariance. Sparsity parameters were chosen for each omic from the sequence [0.01, 0.02, ..., 0.09, 0.1, ..., 0.9], by cross-validation. With this purpose, a balanced dataset, composed of 10 samples per tumor subtype and 10 samples from normal tissue, was randomly taken from the original data, 10 times per each sparsity parameter value. Each time, a simple SGCCA was run, recovering only one component and taking note of the selected number of features and the average variance explained (AVE). Summing the different combinations, in total, every value was tested 11,340 times per omic. Sparsity parameters were chosen in order to obtain the largest AVE with the lowest number of features (Supplementary Figure S1), namely, 0.02 for CpG sites and transcripts and 0.05 for microRNAs.

Data analytics included several stages: independent pre-processing to deal with factors specific to the platforms, while normalization and penalization concern appropriate data integration. Eigenvalue normalization was further performed to equilibrate the still disparate rank of the different values. Separate penalization considers the different signal sizes the distinct omics may have. Shrinking the same CpG coefficients and miRNA coefficients may over-penalize relevant associations yet with effects smaller than those coming from other omics Liu et al. (2018). After the fitting process, we noticed that miRNAs are slightly less penalized than the other omics.

The definite SGCCA for each subtype and the normal tissue was run using the fitted values. The smaller the sparsity value, the fewer features get selected. For each subtype, we used the number of samples minus 1 as ncomp, the default design matrix, and the centroid function, which enables negative correlation.

Feature selection attained by SGCCA is expected to be a bit unstable due to the LASSO penalization. Mimicking the filter used in miRDriver (Bose and Bozdag, 2019), we re-run SGCCA 100 times per subtype, or the normal tissue, using a random subset of half the samples each time. We only kept those features selected at least 70% of the time.

### 2.3 Functional enrichment analysis

SGCCA results include a matrix of the loadings a feature has in each component. The said matrix is quite sparse, except for the features summarizing the relevant information between and within omics. These non-zero loadings indicate co-selected features that can be tested for functional enrichment.

With the idea of exploiting the full set of co-selected features, and not just the transcripts, all the features, being CpG probes, miRNA precursors, or transcripts, were mapped to Entrez gene IDs. Both transcripts and miRNAs have a direct annotation at Entrez, (e.g., hsa-mir-34b becomes MIR34B). To translate CpG probes to Entrez IDs, we recovered the genes affected by each probe from the microarray annotation file. This results in an amplification of CpG representation since one site can be associated with a whole cluster of genes and assumes a methylation effect on overlapping genes, which is not necessarily true. Both are cons of this mapping that need to be considered.

Then, the group of features with non-zero loading in every SGCCA component was submitted to a separate over-representation analysis, taking Entrez IDs as input. Enrichment was run using the clusterProfiler package (Wu et al., 2021) against the pathways from the KEGG database (Kanehisa and Goto, 2000) and against the biological process gene ontology (Consortium, 2021). A significance threshold of FDR-corrected *p*-values < 0.01 was set. The intersection between sets of enriched functions was plotted with the UpSetR package (Gehlenborg, 2019). Functions exclusively enriched in one dataset were tested for over-representation. With this purpose, exclusively enriched functions were grouped according to GOslim and KEGG classes. Dependence between grouped categories and the subtypes was assessed with Fisher’s test, and *p*-values were adjusted for multiple testing using the Bonferroni method.

In an independent manner, we ran a gene set enrichment analysis (GSEA), only with transcript data, to check for functions affected by differential expression. GSEA was also performed with the clusterProfiler package, in this case, without a *p*-value cutoff. The idea is to recover a GSEA enrichment score for every one of the functions over-represented in the SGCCA results. Such scores would answer if functions over-represented among the features related through different omics are also enriched among genes with altered expression. We must stress, however, that all discussed functions are significantly over-represented (*p*-value < 0.01), but only the specified ones also have a significant GSEA score.

### 2.4 Network reconstruction

Chosen functions were represented as networks to draw potentially regulatory models. To achieve this, we estimated mutual information (MI) between every pair of nodes using ARACNE software (Margolin et al., 2006) and then filtered out all the pairs with lower MI than the median value observed for known regulatory interactions. Thus, for each chosen function, we recovered all the features co-selected (co-varying) with the features responsible for the functional enrichment and focused on this set.

1. We extracted a sub-matrix from the original dataset and run ARACNE.
2. We retrieved regulatory interactions involving the selected features. Again, this was performed with the microarray annotation file for the CpGs, assuming position overlap is enough to affect gene expression. The multiMiR package (Ru et al., 2014) was used in the case of miRNAs and TFtargets (github.com/slowkow) for the transcript coding for transcription factors. This latter package queries several resources, namely, TRED, ITFP, ENCODE, TRRUST, and the databases from Neph et al., 2012; Marbach et al., 2016 (Jiang et al., 2007; Zheng et al., 2008; Consortium et al., 2012; Neph et al., 2012; Han et al., 2015; Marbach et al., 2016), which include validated and predicted interactions. We considered those hits coming from ChIP-seq, DNaseI footprinting, and small-scale experiments as validated.
3. We obtained MI values for such regulatory interactions, using the infotheo package (Meyer, 2014) (the use of this specific tool obeys the need to focus on a reduced set of given pairs, instead of estimating all the pairs with a feature of interest in the adjacency matrix, as ARACNE would perform).
4. We took the median MI value for the regulatory interactions as the threshold. Since MI is expected to differ between the distinct kinds of pairs, different thresholds were obtained for the different types of edges: CpG–transcript, CpG–miRNAs, TF transcript–transcript, and miRNA–transcript. The median was preferred over the mean to avoid outliers dominating the threshold.
5. The MI value distribution obtained with ARACNE was contrasted between types of edges, *via* Kolmorogov–Smirnov tests. If distributions were not significantly different, the lowest median MI from regulatory interactions—obtained with infotheo—was chosen as the unique threshold to pass, no matter the edge-type, relaxing the threshold and increasing the MI interactions accepted in the final network.

The output of these items is a table with predicted interactions and weights that illustrate the largest statistical dependencies between the features selected by the SGCCA.

### 2.5 Network analysis

Mutual information networks were analyzed with the igraph package (Csardi and Nepusz, 2006) and represented with Cytoscape (Shannon et al., 2003), making use of the RCy3 package (Gustavsen et al., 2019).

Node colors represent logFC values between every subtype and the normal tissue. MiRNA differential expression went through *voom* normalization and *eBayes* limma function. Since the batch effect was not corrected in methylation data, we used the missMethyl package for the differential analysis. This tool removes systematic errors of unknown origin, bypassing the lack of batch-effect correction (Maksimovic et al., 2015).

The node degree was calculated for the whole network; however, only those network components with features annotated as players of a function are shown in the corresponding figures. Since Her2+ and luminal B subtypes produce large networks, we further zoomed in the graph by selecting only the first neighbors of functional features. Such subnetworks may serve as a model of the regulatory pressures influencing the function.

Every neighbor of a functional node was searched in PubMed, together with the associated functions, to find out if some biological role has already been suggested. PubMed was also queried with every pair of interacting nodes, as well as the databases for predicted regulatory links accessible through multiMir. Transcription factor-related features are identified according to the list from humantfs (Lambert et al., 2018). This achieves a fairly automated way to build a regulatory model for the functions enriched in the SGCCA.

## 3 Results and discussion

By applying SGCCA, we have identified, for each one of the breast cancer subtypes, transcripts whose expression patterns better reflect the variance in its own block, while also co-varying with the other blocks of data. The pattern of selected features by omics and subtype is provided in Supplementary Figure S2.

SGCCA uses a LASSO penalization, which may select inconsistent sets of features. Since this could affect the reliability of functional enrichment, identifying functions dependent on unstable features, we just proceeded with the features most consistently selected, whose proportion is shown in Supplementary Figure S3. There are no individual transcripts or miRNAs selected simultaneously across all five datasets, but there are six CpG sites in this situation which potentially affect MAPK8IP3, AFAP1, LFNG, and VSTM2B.

The transcripts repeatedly selected in the same subtype have known associations with breast cancer. The top three transcripts selected more often for the basal subtype are MCL1, CTNNA1, and NOTCH3. MCL1 is an anti-apoptotic member of the BCL2 family that is required for mammary stem cell function (Fu et al., 2015), and it is expected to be overexpressed in tumors of this subtype (Farrugia et al., 2015). Meanwhile, catenin alpha 1 is postulated to act as a tumor suppressor in E-cadherin-negative basal-like breast cancer cells (Piao et al., 2014), and NOTCH3 seems to function as a promoter of the epithelial–mesenchymal transition (Liang et al., 2018).

Her2 enriched has also been clearly associated with its most selected transcripts: CEACAM5, ACACA, and PGK1. Though heterogeneously expressed, Her2-enriched tumors tend to be positive for CEACAM5 (Bechmann et al., 2020) and so this adhesion molecule has been suggested as a target for T-cell bi-specific antibodies (Messaoudene et al., 2019). Inhibitors of acetyl-CoA carboxylase, ACACA, work over MCF-7 cells overexpressing Her2 by interfering with cancer stem cell lipid biosynthesis and the Warburg effect (Corominas-Faja et al., 2014). At last, PGK1 protein has been found overexpressed in these tumors (Schulz et al., 2009), while being linked to macrophages and stratifying patients at higher risk (Li et al., 2021).

Interestingly, microRNAs from the let-7 family were among the top selected for basal, Her2+, and luminal B subtypes, as well as for normal breast tissue. These miRNAs regulate JAK-STAT3 and Myc signaling pathways, thus affecting stemness and metastasis (Thammaiah and Jayaram, 2016).

### 3.1 Functions enriched on SGCCA output differ between datasets

After inspecting the overall output of SGCCA, we wanted to know if there are functions involving the co-varying features. Enrichment against GO biological processes and KEGG pathways allows us to identify functions affected by the specific regulatory mechanisms identified.

**FIGURE 2:**
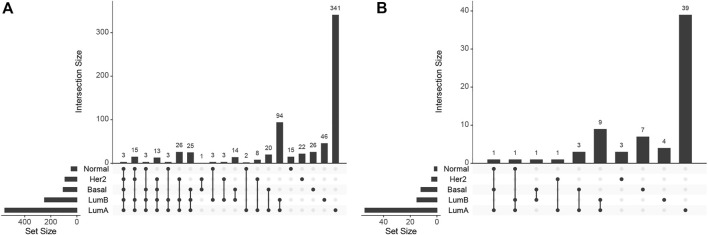
UpSet plot for **(A)** biological processes and **(B)** KEGG pathways enrichment.

A total of 683 GO biological processes and 69 KEGG pathways were found significantly over-represented (FDR adjusted p. value < 0.01) among the SGCCA co-selected features. Figure 2 shows the intersections between subtypes. Few functions were found enriched across all subtypes, and most of them are either exclusive or shared only by a pair of subtypes. That is, functions associated with DNA methylation and miRNA expression are not the same for all subtypes.

There are three biological processes significantly enriched (FDR-corrected *p*-value ≤.0099, for the specific values, see Supplementary Table S1) in the four subtypes and the normal tissue. These are the developmental processes: metanephric nephron development (GO:0072210), metanephros development (GO:0001656), and pattern specification process (GO:0007389). Since GO:0072210 is a part of GO:0001656, they may be considered the same.

Then, we wondered if functions linked with DNA methylation and miRNA expression in cancer and normal tissue maintain an intact circuitry connecting CpGs, transcripts, and miRNAs. In more general terms, does a function enriched twice involve identical features and interactions?

### 3.2 Features responsible for the same functional enrichment differ across subtypes

**FIGURE 3:**
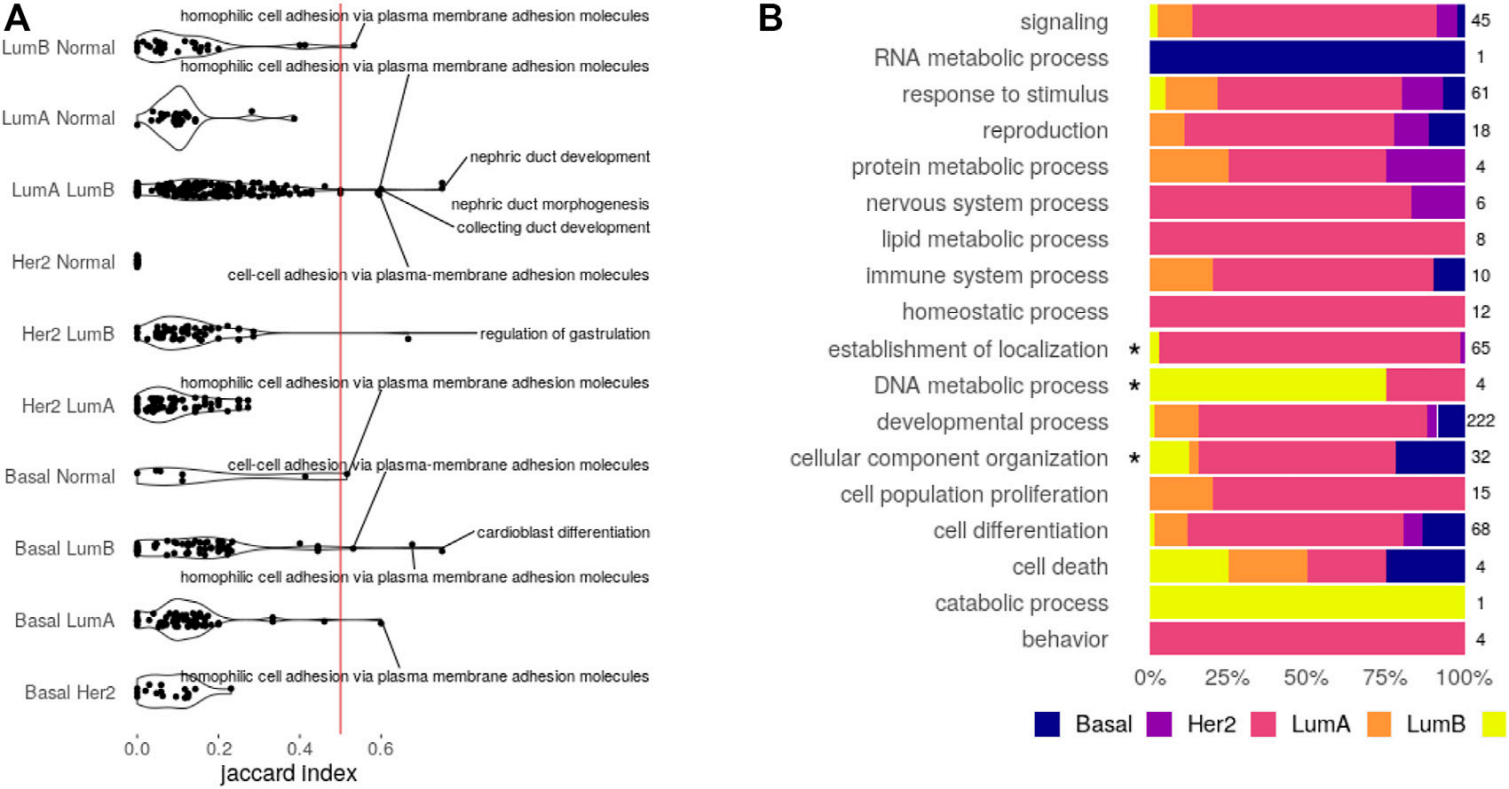
Enriched functions. **(A)** Feature similarity between functions shared by the pair of datasets indicated. Functions with similarity over 0.5 are displayed. **(B)** Bias of exclusive functions. An asterisk marks categories with significant over-representation (Fisher’s test, Bonferroni adjusted *p*-value < 0.05).

The first step toward a shared circuitry connecting CpGs, transcripts, and miRNAs in different phenotypes would be to have the same (or similar) features behind the functional enrichment. To verify if this happens, we calculated the Jaccard index for every pair of functions enriched more than once. The Jaccard index divides the size of intersection between two sets by their union, measuring similarity with a normalized value between 0 (fully disjoint sets) and 1 (the same set). Distributions for the Jaccard index are shown in Figure 3A.

The obtained distributions are enough to state that, for most functions, the CpG–transcript–miRNA circuitry is not the same across datasets since the features involved are not the same. Only seven biological processes enriched in a given pair of SGCCA results share more than 50% of the involved features. Five of them are related to development, while the other two are related to cell adhesion. These are the functions that may share the interactions between CpG sites, transcripts, and miRNAs.

If this index hints at the similarity between subtypes pertaining to CpG–transcript–miRNA co-variation, the distance with Her2-enriched subtype results are intriguing. This may be caused by a bias induced by the low number of samples. Or perhaps this is associated with the lower correlation with DNA methylation patterns (Network et al., 2012). Not surprisingly, the pair with the most similarly enriched functions corresponds to the two luminal subtypes.

### 3.3 Exclusive category over-representation

To answer if functions exclusively found in one dataset bring to light subtype-specific properties, we analyzed over-representation of GOslim categories and KEGG classes. The proportion of biological processes found for each dataset in every one of the categories is given in Figure 3B, while the equivalent plot for KEGG pathways is found in Supplementary Figure S4.

None of the KEGG classes is biased toward a given subtype, but there is an enrichment for the categories: *cellular component organization* in the basal SGCCA components, *establishment of localization* in luminal A, and *DNA metabolic process* in the normal tissue. There are seven biological processes behind the *cellular component organization* over-representation, comprising five processes related to axon extension, which are clustered with regulation of the extent of cell growth. Collagen fibril organization is not in the cluster and is the seventh process, suggesting a potential bond between the basal subtype and invasiveness.

In the case of luminal A, there are 62 biological processes behind the over-representation of *establishment of localization*. These processes affect transport and secretion and conform to 32 different clusters. Regarding over-representation in the normal tissue, it is interesting that it is related to DNA alkylation and methylation processes, perhaps implying that these processes are somehow disarranged on the tumor subtypes.

### 3.4 Within subtypes, different functions can be connected through correlated features

When checking the features responsible for the enrichment of a given function, we discovered that several functions are enriched in the exact same set of co-varying features, that is, the same set of SGCCA components. This suggests some level of crosstalk between functions that can be connected through correlated features. This observation has been made subtype-wise and implies that a single network of correlated features may actually span several functions.

**FIGURE 4:**
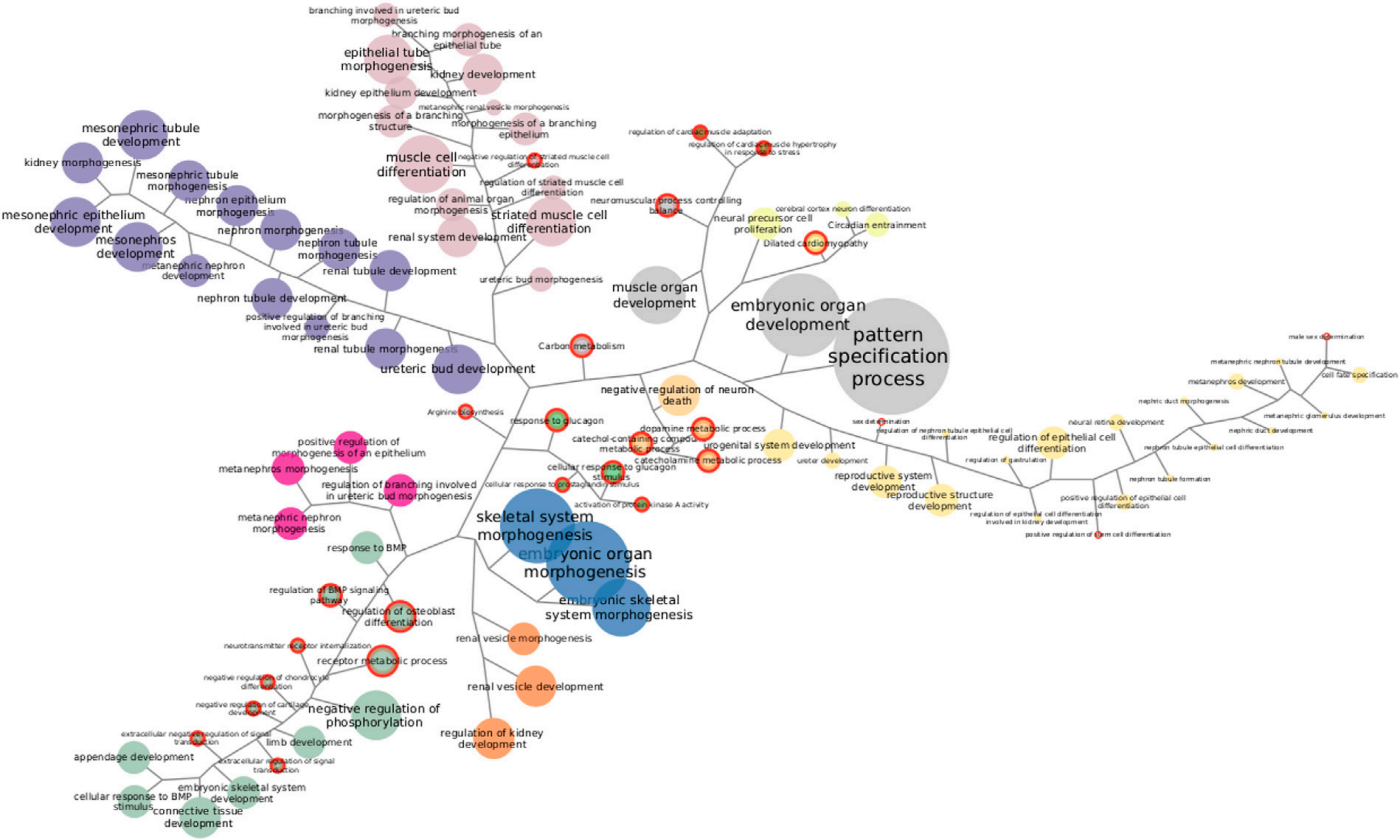
Functions enriched in Her2 SGCCA components. Both KEGG pathways and GO biological processes are represented together. Same-color clustered nodes are enriched in the same components. Nodes in gray do not belong to any cluster. The size of nodes and labels reflect the number of features behind the enrichment. Functions exclusively found in this subtype are highlighted with a red border.

Going through each subtype separately, we clustered functions by the proportion of SGCCA components shared. Figure 4 shows Her2 clusters. There are 11 clusters and six functions that cannot be grouped since they involve features that are not related with the clusters. Taking the bigger labels as a guide, purple, orange, and fuchsia clusters are related with development of kidney structures. Green and blue clusters at the bottom are linked with connective tissue development. Pale pink nodes refer to distinct processes of morphogenesis, while the nodes in yellow allude development of reproductive structures. The small brown and pale green clusters are related to cardiac muscle and neural cells, respectively. Finally, the small clusters in the center, in bright green and pale orange, are linked with metabolism and loaded with functions exclusively found in this subtype, a fact that may be interesting to explore further. The functions enriched with the most genes do not form a part of any cluster.

Clustering exposes information that needs to be accounted when discussing one particular enrichment. Functions exclusively found in one subtype may reveal mechanistic explanations of subtype-specific alterations, but, if exclusive functions are clustered with others that are non-exclusive and better represented, relevance may be debatable. Similarly, clusters may help explain some odd enrichments, like the one found in the luminal A dataset for morphine addiction. Morphine addiction has been found enriched on methylation-driven genes (Xu et al., 2019) but depends on features correlated with those responsible for ECM–receptor interaction, suggesting co-variation may be pulling up the enrichment for this addiction. Even after considering clusters, there are enrichments hard to figure out fully; however, some specific features can be actually tracked (Supplementary Tables S1, S2).

In order to select functions to explore further, we repeated the analysis described with Her2+ for each SGCCA result. While not all clustering are displayed here, full groups and enrichment results are supplied as Supplementary Files. A filtering step was necessary because, even with the clustering, there are almost 500 sets of functionally related features. It is interesting that the two cell adhesion processes with the Jaccard index over 0.5 appear consistently out of any cluster in the subtypes with such enrichment.

### 3.5 Network examples

In our path to answer if a function enriched twice involves identical features and interactions, we found that a given function is commonly enriched through distinct sets of features in two different datasets. At the same time, we observed several functions over-represented among the same sets of co-selected features and wondered how functions were connected. Functions involving the same features are already identifiable in the annotation databases, but by means of this multi-omic integration strategy, we have been able to find cross-linking paths across single layers and maybe even connect seemingly independent functions through multi-omics pattern co-variation. To check how this appears, we built mutual information (MI) networks. The networks went through a stringent threshold to keep just the interactions that are most likely regulatory. To this end, we obtained the MI values accompanying true regulatory interactions and took the median value as the minimum MI required to consider an edge as possibly regulatory. Within these reduced sets of interactions, the following figures show the network components that contain those features annotated as participating in the functions, though some of the obtained networks extend further.

The intuition is that co-selected features, whose patterns are correlated with those of functional features, may also be participating in a given function. Beyond that, nodes for miRNAs, CpGs, and transcripts that ultimately code for transcription factors may be playing regulatory roles. The stringent threshold attempts to filter out the interactions owed to simple co-variation. Two broad possible scenarios are expected, 1) disconnected components per function, each with its own potential regulators, or 2) functions that crosstalk through common features, whose potential regulators could be of medical interest. The different scenarios are exemplified through the four subtypes and the normal tissue in the coming sections.

#### 3.5.1 HIF-1 signaling in the basal subtype

Hypoxia-inducible factor 1 (HIF-1) signaling is one of the KEGG pathways enriched exclusively in the basal SGCCA results. HIF-1 is the master regulator of oxygen homeostasis since it induces transcription from at least 100 hypoxia-responsive elements (Corrado and Fontana, 2020). HIF-1 signaling is activated in tumors not only under hypoxic conditions but also by oxygen-independent factors, like TP53 and BRCA mutations (de Heer et al., 2020), which have been associated with the basal subtype (Network et al., 2012).

**FIGURE 5:**
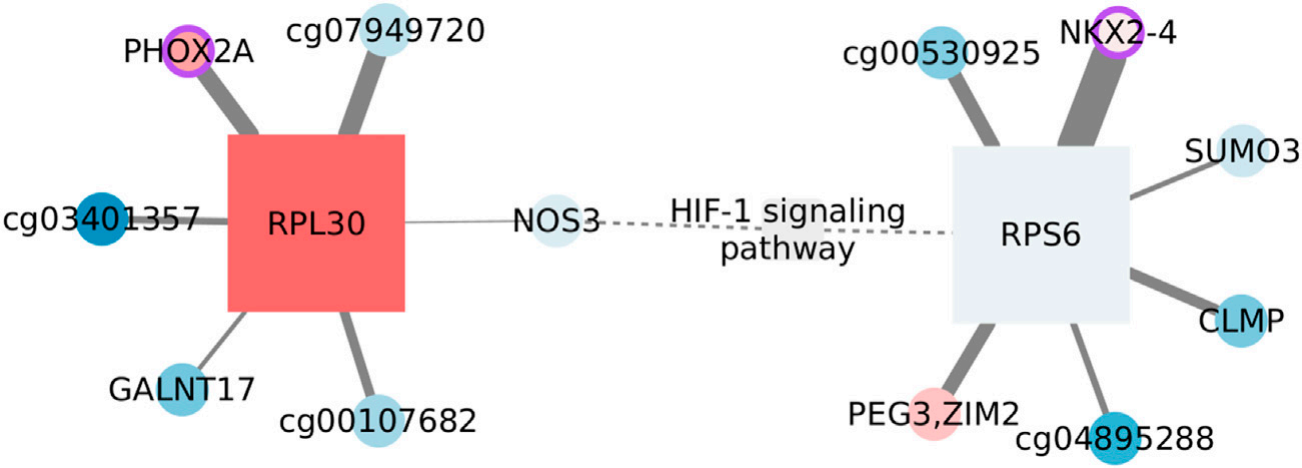
Features connected with HIF-1 signaling in the basal subtype. Circles represent CpGs, and squares are transcripts. When possible, CpGs are identified with the symbol of the gene they affect; otherwise, the ID of the probe is used. The shades of red indicate the level of overexpression/methylation against the normal tissue, while blue tones represent values under what is expected. The node size reflects its degree. A purple border identifies nodes whose protein plays a transcription factor role. The weight of the link is the extent of mutual information between connected nodes. Dashed edges link MI components with prior information.

The network we identified for this function is given in Figure 5. AMPK signaling is enriched in a subset of the same SGCCA components such as HIF-1 signaling, which is consistent with the idea that these two pathways interplay in cancer metabolism re-programming (Moldogazieva et al., 2020). However, after applying the MI threshold, each pathway occupies disconnected components.

Only two functional features, that is, annotated as participants of the function, pass the MI threshold, NOS3 and RPS6. It is important to clarify that the enrichment does not rest only on these two features, but we only find interactions over the threshold for them. There are also two nodes that have been linked with the signaling pathway without being participants as such. PEG3 gets upregulated after hypoxia in mouse lungs (Wollen et al., 2013), while SUMO3 would be one of the modifiers affecting HIF-1 stability (Matic et al., 2008). Thus, nodes seem to be associated with the function.

On the other hand, the complete network is formed by CpG–transcript interactions, more specifically, by edges linking a CpG with a transcript coding for a ribosomal protein. Since CpG sites are not in the same chromosome as the transcript, a direct regulatory influence can be discarded. To account for indirect relations, we estimated the mutual information between the corresponding transcripts, even when these were not originally in the SGCCA set of co-selected features. Obtained MI values are smaller than the global threshold and smaller than the edges between CpGs and ribosomal protein-coding transcripts. Hence, indirect effects going through the transcript linked with the CpG do not seem to fully explain the phenomenon.

Most nodes are not significantly different from the normal tissue, either regarding expression or methylation values. This is consistent with the lack of significance of the pathway GSEA score (NES = 0.9252, adjusted *p*-value: 0.7937). HIF-1 signaling in the basal subtype is transcriptionally comparable with that of the normal tissue. Nevertheless, the pathway is not found enriched in the normal tissue SGCCA output, suggesting a change in the correlation between omics.

#### 3.5.2 Positive regulation of stem cell differentiation in the Her2-enriched subtype

**FIGURE 6:**
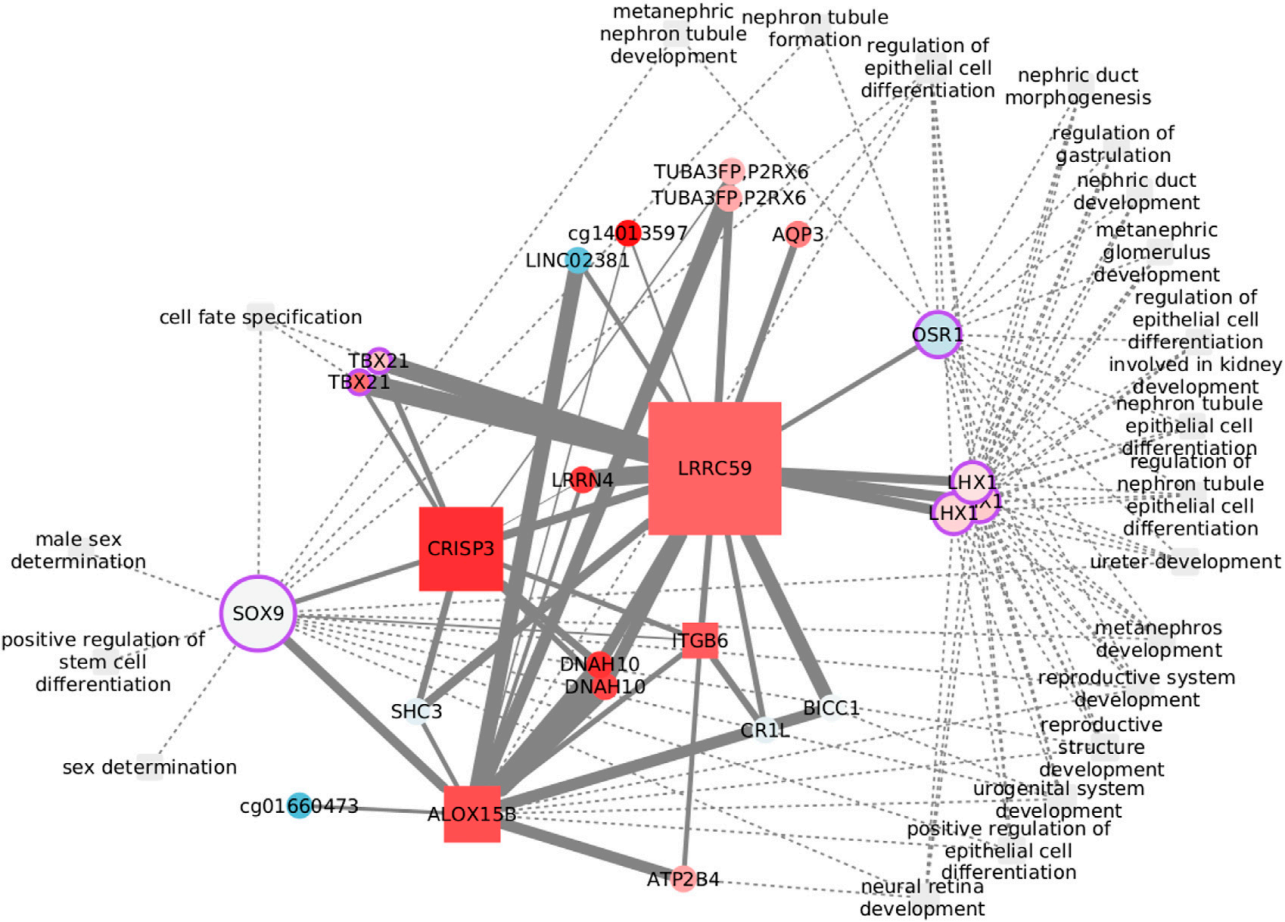
Features connected with the regulation of stem cell differentiation in the Her2-enriched subtype. Node size reflects the betweenness centrality.

Cancer stem cells are largely responsible for relapse and metastasis. Her2 variants, observed in Her2+ patients with poor clinical outcomes, have been reported to drive maintenance and enrichment of breast cancer stem cells (Pupa et al., 2021). *Positive regulation of stem cell differentiation* was found enriched exclusively in Her2+ data, but related processes also appear in the other three subtypes. The process is clustered with several other functions, as shown in Figure 6, where we have focused on the first neighbors of the functional features.The transcription factor SOX9 is the only feature from *positive regulation of stem cell differentiation* with edges passing the MI threshold. SOX9 binds functions related with cell fate and sex determination, while LHX1 and OSR1 are at the crossroads of most functions. None of the edges has been previously reported, but several nodes have known links with these functions. The relation between CRISP3 and sex determination, for instance, may be explained by the role of the protein in sperm function (Weigel Muñoz et al., 2019) and its up regulation in prostate cancer (Pathak et al., 2016). DNAH10 is another feature with a known bond with sex determination, specifically with sperm flagella morphological abnormalities (Li et al., 2022). The connection with ITGB6 is perhaps weaker since it rests only on differential expression analysis of prostate cancer (Li et al., 2013). CR1L is involved in B lymphocyte activation (Fernández-Centeno et al., 2000) and may have a role in renal injury (He et al., 2005). Finally, the somehow unexpected *neural retina development* is related with the function of SHC3 (Nakazawa et al., 2002).

The functional implications of some of these nodes are specifically dependent on DNA methylation. Although epigenetically altered CR1L is linked with Alzheimer’s and dementia (Bahado-Singh et al., 2021), DNAH10 has emerged when studying renal carcinomas with a CpG-island methylator phenotype (Arai et al., 2015). Finally, CpG methylation of the lncRNA LINC02381 functions as a tumor suppressor in colorectal cancer (Jafarzadeh et al., 2020). While all of these features are represented by CpG sites in the network, LINC02381 appearance highlights the complexity of transcription regulation and the need to widen multi-omic analysis to include more data layers.

Despite that transcription factors may be the obvious option to explore the crosstalk between biological processes, less explored options, like ALOX15B, CRISP3, and LRRC59, with elevated graph betweenness, may result of interest.

#### 3.5.3 Ras signaling pathway in the luminal A subtype

Ras signaling is one of the many pathways exclusively found enriched in the luminal A subtype. It is a well-documented pathway influencing cancer aspects like cell proliferation, survival, migration, and differentiation. Although the pathway is more frequently activated in the other subtypes, it has been reported as an indicator of poor prognosis in luminal tumors (Wright et al., 2015). Not surprisingly, Ras signaling components are under-expressed relative to the normal tissue (NES: 1.5796, adjusted *p*-value: 0.0084) in this analysis.

Only one functional feature endures the MI threshold, GNB2. The subunit beta 2 of G protein links the signaling pathway with a set of CpGs associated with cell communication and brain function, through the calcium sensor SYT13. Genes affected by the CpGs include the brain active kinase, STK32C; MIDN, that is predicted to enable kinase binding; OBP2B, which is supposed to enable binding of small volatile molecules; a TF from early brain development, RFX4; and SYCN, which is predicted to be active in exocytosis.

Among the remaining nodes, the connection with the CUX1 CpG site agrees with the cooperation observed between this transcription factor and Kras-G12V mutant in lung cancer (Ramdzan et al., 2014). In a similar way, PTBP1 overexpression is known to co-occur with oncogenic KRAS mutations in colon cancer (Hollander et al., 2016). Finally, a connection with the transferrin receptor internalization protein, SH3BP4, has been predicted before by a random forest classifier (Xin et al., 2021).

**FIGURE 7:**
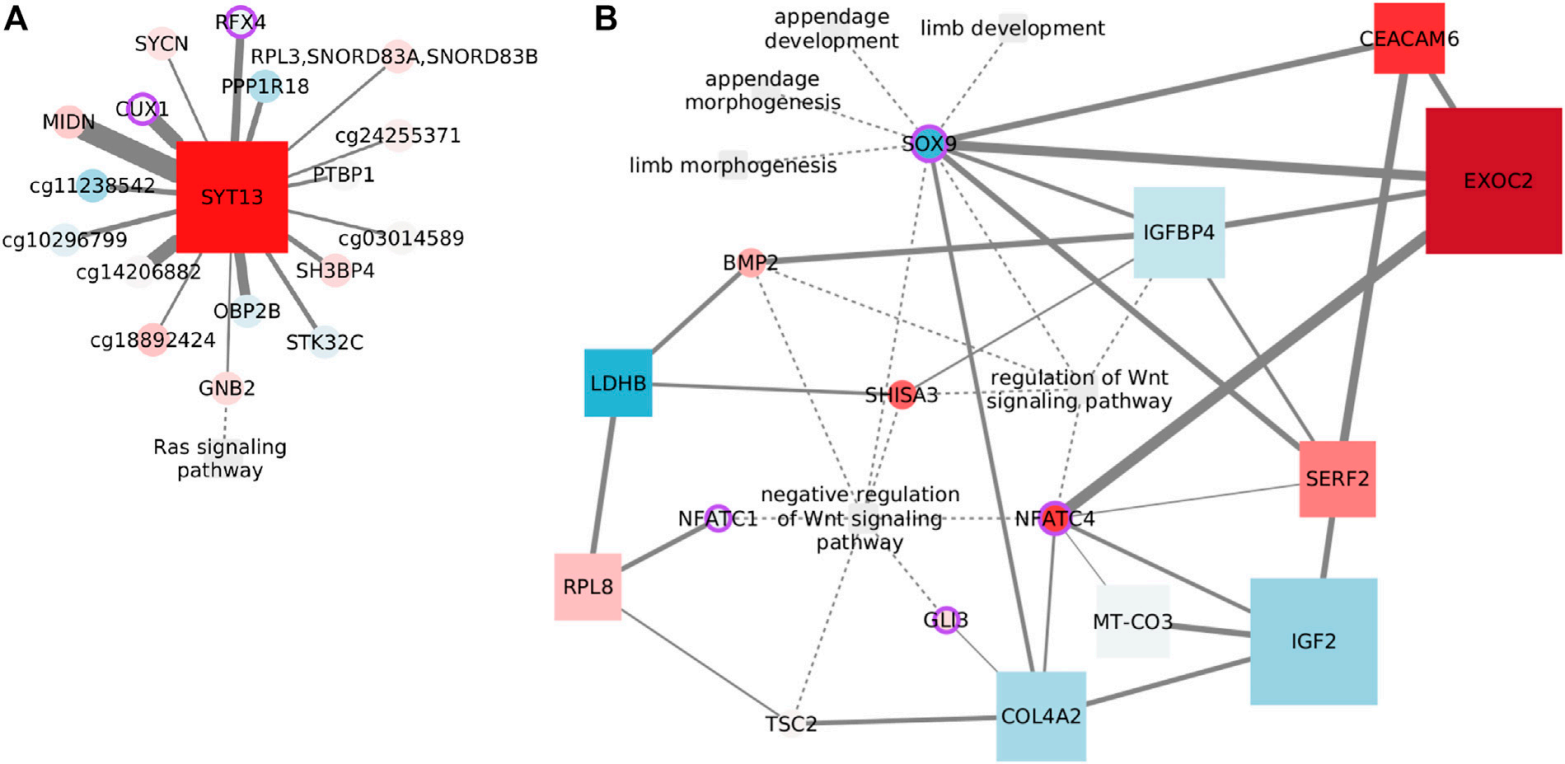
Example networks for the luminal subtypes. **(A)** Features connected with the Ras signaling pathway in luminal A data. Node size indicates degree. **(B)** Features connected with Wnt signaling in the luminal B subtype. Node size reflects betweenness centrality.

Again, the network shown in Figure 7A links a transcript with CpG sites all over the genome. Although it has been proposed that Ras signaling controls aberrant DNA methylation (Patra, 2008), the specific influence nodes may have over the signaling pathway remains unclear.

#### 3.5.4 Negative regulation of the Wnt signaling pathway in the luminal B subtype

Wnt signaling normally controls organ development. In breast cancer, Wnt signaling is involved in tumor proliferation and metastasis, immune microenvironment regulation, stemness maintenance, and therapeutic resistance (Xu et al., 2020). The relevance of this function does not end here, but it has also been associated explicitly with the luminal B subtype. Though generalized DNA hypomethylation is common in cancer (Vidal Ocabo et al., 2017), a fraction of luminal B tumors exhibit hypermethylation, specifically affecting Wnt signaling (Network et al., 2012).

In our results, *negative regulation of* the *Wnt signaling pathway* is exclusively found enriched in this subtype, but related Wnt pathways were also found for luminal A. The cross-talking functions shown in Figure 7B are not in the same cluster but are found in a subset of the SGCCA components, where negative regulation of Wnt appears. Since these related functions makeup the largest network—after the threshold—we have, and this network consists of a large single component, we decided to focus on the first neighbors of the functional features.

As expected, *negative regulation of Wnt signaling* and *regulation of Wnt* share functional features. The transcription factor for skeletal development, Sox9, is represented by its CpG at the crossroad between Wnt signaling with the developmental processes, but there are also multiple indirect paths. Since the genes coding collagen subunit Col4a2 and cell adhesion molecule Ceacam6 are targeted by Sox9 (Sumi et al., 2007), and Sox9 acts in cooperation with Gli3 (Tan et al., 2018), that pair of edges are easy to justify. Similarly, the link between COL4A2 and NFATC4 could be explained by the inhibition of the nuclear translocation of NFATc4 by Col4a2 in cardiomyocytes (Sugiyama et al., 2020), while both COL4A2 and IGF2 code for extracellular proteins deregulated under diseases with EMT (Bueno et al., 2011). Additionally, bone marrow stromal cells induced with IGFBP4, among other factors, overexpress SOX9 (Liu et al., 2012). Insulin-like growth factor-binding protein 4 is also connected with BMP2, as IGFBP4 overexpression impairs BMP2-induced osteogenic differentiation (Wu et al., 2017).

In summary, there are sound biological reasons to expect co-variation of the connected features. The question to solve is how such connections affect Wnt signaling and luminal B cancer progression, specifically what is the role of the node with the highest betweenness. Exocyst complex component 2 is related with the Wnt pathway as an effector of Hedgehog signaling (Arraf et al., 2020) and has been associated with metastasis and different cancer types (Cerhan et al., 2014; Hazelett and Yeaman, 2012; D’Aloia et al., 2018), but not with breast cancer.

#### 3.5.5 DNA methylation in the normal adjacent tissue

DNA methylation is exclusively enriched in the normal tissue, but we choose to discuss it because of its relevance for cancer (Baylin and Jones, 2016). In addition, unlike the other examples, this network does contain microRNAs, including the top selected let-7a-2.

**FIGURE 8:**
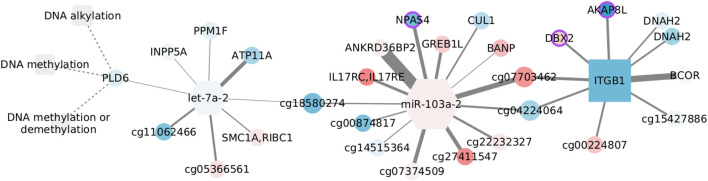
Features connected with DNA methylation in the normal adjacent tissue. Color corresponds to log fold changes in the basal subtype.

For consistency, we colored the nodes in Figure 8. However, since the normal tissue is our reference value, we used the log fold changes obtained by contrasting basal and normal tissue expression. This subtype has significant overexpression of related genes (NES = 1.9251, adjusted *p*-values = 0.0031) and has been linked with hypomethylation (Network et al., 2012). Yet, we have to emphasize that DNA methylation is not enriched in the basal data, and so, the relation between CpGs, miRNAs, and transcripts may not follow what is suggested in this figure.

Despite none of the interactions has been reported, a couple of nodes are somehow connected with the DNA methylation machinery. AKAP8L interacts with core subunits of the H3K4 histone methyltransferase complexes (Bieluszewska et al., 2018), whose action is interrelated with DNA modification (Rose and Klose, 2014). BCOR is part of the non-canonical polycomb repressive complex 1 and is altered in distinct cancer types (Astolfi et al., 2019). It has been observed that BANP can open the chromatin at unmethylated CpG-island promoters, thus activating essential genes in pluripotent stem and differentiated neuronal cells (Grand et al., 2021). Finally, *de novo* DNA methyltransferase, DNMT3b, can interact with CUL1, involving this node in aberrant methylation (Shamay et al., 2010).

In contrast, another set of nodes hinges on epigenetic silencing, as is the case of INPP5A in lung adenocarcinoma (Ke et al., 2020). Together with ATP11A, INPP5A CpG methylation has shown discriminatory capacity for colorectal cancer (Izquierdo et al., 2021). In the same manner, ATP11A methylation distinguishes several diseases including metastatic-lethal prostate cancer (Zhao et al., 2017), while a methylation signature including the growth regulation by estrogen in breast cancer 1 like GREB1L separates gastric adenocarcinoma cases by overall survival, and DBX2 methylation marks the serum from hepatocellular cancer patients (Zhang et al., 2013). Similar to its paralog DNAH10, DNAH2 aberrations are frequent in renal carcinomas with a CpG-island methylator phenotype (Arai et al., 2015). Although unexpected, the brain-specific transcription factor NPAS4, present in the form of a CpG site, is known to be regulated by DNA methylation (Furukawa-Hibi et al., 2015) and has been linked with colon adenocarcinoma survival (Luo et al., 2021). Last, though ITGB1 methylation is expected to be constant both in cancer and normal tissue (Strelnikov et al., 2021), alteration of the gene expression has been observed in basal-like tumors and cells with BRCA mutation, highlighting the relevance of migration and mesenchymal properties for this subtype (Privat et al., 2018).

Interestingly, the two miRNAs in the network are associated with migration and invasion, although in opposite ways. The let-7 family works as a tumor suppressor and is inhibited by DNA methylation and several regulators (Thammaiah and Jayaram, 2016). Contrastingly, miR-103 acts as an oncogene in triple-negative tumors, and its over-expression is linked with poor prognosis (Xiong et al., 2017). In spite of the low fold changes, the expression of both miRNAs is coherent with what would be expected in the basal subtype.

## 4 Conclusion

Here, we have described the kind of multi-omic network models that can be obtained through the sequential application of SGCCA and ARACNE. The collection of interactions shown in any of these networks suggests a multi-omic model that may or may not have regulatory implications. To asseverate regulation, wet laboratory testing would be needed. However, the nature of nodes as CpG sites, microRNAs, or transcript coding for functional proteins must be considered, as shown in the examples. Although further testing is required, the examples embody the level of details we can get in the way toward targeted experimental validation of multi-omic regulatory phenomena.

Though the interactions encountered seem to be subtype-specific, given the low values of the Jaccard index, there is no restriction to believe these same associations could not be repeated in other contexts, with somehow equivalent patterns of methylation and expression. Instead, an interesting question arises about the traceability of tissue and disease signals. A fair attempt to carry out would be to compare cancer and tissue networks with the same nodes, even if the edge weights are disparate, which were not produced here. Also, it has to be noticed that the normal adjacent tissue may not be the best control since it carries detected alterations across tissues (Aran et al., 2017).

The use of SGCCA allowed us to identify the functions enriched in features co-varying across DNA methylation, transcript, and miRNA expression. This does not mean such functions may not be influenced by other regulatory mechanisms: this simply indicates the functions, like HIF signaling in the basal subtype, depending the furthest on features whose methylation and expression co-vary. The con of the method is the instability of the LASSO, which forced us to keep just the features identified in over 70% of subsamples. Even when other tools (Hernández-de Diego et al., 2018; Meng et al., 2019) could achieve the multi-omic functional enrichment without the instability issue, we prefer the sparse method exactly because of the stable portion of the feature set. Then, possible improvements include the elastic network penalization, which overcomes the stability problem.

mixOmics output for the SGCCA includes a complete graph connecting all the features selected in a component. However, having found the same functions over-represented in different components, we wanted to further explore the relations among all the features co-varying with those associated with a given function. The mutual information statistical dependency measure has desirable properties for multi-omic integration, such as being able to capture non-linear relations and being a parameterization invariant. Moreover, we wanted to discern likely regulatory interactions, a task that has been successfully achieved with ARACNE for transcriptomics. With edges linking different types of nodes, such discerning becomes harder because ARACNE’s data processing inequality (DPI) cannot be used in a straightforward manner. Thus, the setting of varying thresholds based on regulatory interactions is established. In this case, MI ability to recover non-linear relations may not be fully profited, being posterior to the lineal filter of SGCCA. MI is, however, used as a way to bring together all the results concerning a function and highlight some potentially interesting pairs of nodes.

The DPI posed with ARACNE discards the lowest weighted edge from a triad, as a likely indirect interaction driven by the other pair of nodes. The difficulty of using it comes from the observation that mutual information distributions change with the different omics (Drago-García et al., 2017). While maintaining the treatment of lower weighted interactions as indirect, the threshold we applied accounts for the difference between omics by estimating MI values from known regulatory interactions.

It is worth considering that MI has a dependency on the number of observations, which varies between subtypes and the normal tissue. Her2 enriched has a smaller number of samples than recommended, and so special care must be taken with it. Given that MI is rank-invariant, it is expected that, even with the stringent threshold, only a subset of the interactions in Figure 6 keep relevance when increasing dataset size. By progressing from a set where every feature is correlated with one another to highly significant interactions (Pethel and Hahs, 2014; Mukherjee et al., 2020), we pursue an automatic assembly of regulatory models. Tools better suited to find regulatory interactions (Kuijjer et al., 2020; Sonawane et al., 2021) require prior information not always available or heavier calculations (Weighill et al., 2021), making the approach described here an accessible solution.

To end with the pros and cons’ discussion, here, we have overlooked interactions between CpG sites because those are beyond described regulatory mechanisms. Nevertheless, links between CpG sites are accompanied by large MI values that would surpass our threshold and may become of relevance in the cancer context (Akulenko and Helms, 2013; Zhang and Huang, 2017). On the other hand, links with miRNAs were expected but only appeared in the normal tissue example. Drago-García et al. had already reported lower MI values for these types of links (Drago-García et al., 2017). Despite the threshold attempted to incorporate this difference on the MI, our multi-omic pipeline does not recover miRNA interactions as well as other dedicated methods (Bose et al., 2022).

The networks produced in this way capture statistical dependencies that may guide further work. However, such a hypothetical future work depends on a user being able to find these kinds of networks and research the reasons behind a statistical dependency. Article databases can serve this purpose, as we have done here, but may become unspecific. Instead, network databases (Arif et al., 2021; Ben Guebila et al., 2022) may offer a smoother connection between wet and dry laboratories, in order to transcend statistical description toward actual knowledge acquisition.

## Data Availability

The original contributions presented in the study are included in the article/Supplementary Material, further inquiries can be directed to the corresponding author.

## References

[B1] Akulenko R. Helms V. (2013). Dna co-methylation analysis suggests novel functional associations between gene pairs in breast cancer samples. Hum. Mol. Genet. 22, 3016. 10.1093/hmg/ddt158 23571108

[B2] Arai E. Gotoh M. Tian Y. Sakamoto H. Ono M. Matsuda A. (2015). Alterations of the spindle checkpoint pathway in clinicopathologically aggressive c p g island methylator phenotype clear cell renal cell carcinomas. Int. J. cancer 137, 2589–2606. 10.1002/ijc.29630 26061684 PMC4755138

[B3] Aran D. Camarda R. Odegaard J. Paik H. Oskotsky B. Krings G. (2017). Comprehensive analysis of normal adjacent to tumor transcriptomes. Nat. Commun. 8, 1077–1114. 10.1038/s41467-017-01027-z 29057876 PMC5651823

[B4] Arif M. Zhang C. Li X. Güngör C. Çakmak B. Arslantürk M. (2021). Inetmodels 2.0: an interactive visualization and database of multi-omics data. Nucleic acids Res. 49, W271–W276. 10.1093/nar/gkab254 33849075 PMC8262747

[B5] Arraf A. A. Yelin R. Reshef I. Jadon J. Abboud M. Zaher M. (2020). Hedgehog signaling regulates epithelial morphogenesis to position the ventral embryonic midline. Dev. Cell. 53, 589–602. 10.1016/j.devcel.2020.04.016 32437643

[B6] Aryee M. J. Jaffe A. E. Corrada-Bravo H. Ladd-Acosta C. Feinberg A. P. Hansen K. D. (2014). Minfi: A flexible and comprehensive bioconductor package for the analysis of infinium dna methylation microarrays. Bioinforma. Oxf. Engl. 30, 1363–1369. 10.1093/bioinformatics/btu049 PMC4016708 24478339

[B7] Astolfi A. Fiore M. Melchionda F. Indio V. Bertuccio S. N. Pession A. (2019). Bcor involvement in cancer. Epigenomics 11, 835–855. 10.2217/epi-2018-0195 31150281 PMC6595546

[B8] Bahado-Singh R. O. Vishweswaraiah S. Aydas B. Yilmaz A. Metpally R. P. Carey D. J. (2021). Artificial intelligence and leukocyte epigenomics: Evaluation and prediction of late-onset alzheimer’s disease. PloS one 16, e0248375. 10.1371/journal.pone.0248375 33788842 PMC8011726

[B9] Baylin S. B. Jones P. A. (2016). Epigenetic determinants of cancer. Cold Spring Harb. Perspect. Biol. 8, a019505. 10.1101/cshperspect.a019505 27194046 PMC5008069

[B10] Bechmann M. B. Brydholm A. V. Codony V. L. Kim J. Villadsen R. (2020). Heterogeneity of ceacam5 in breast cancer. Oncotarget 11, 3886–3899. 10.18632/oncotarget.27778 33196697 PMC7597409

[B11] Ben Guebila M. Lopes-Ramos C. M. Weighill D. Sonawane A. R. Burkholz R. Shamsaei B. (2022). Grand: A database of gene regulatory network models across human conditions. Nucleic acids Res. 50, D610–D621. 10.1093/nar/gkab778 34508353 PMC8728257

[B12] Bersanelli M. Mosca E. Remondini D. Giampieri E. Sala C. Castellani G. (2016). Methods for the integration of multi-omics data: Mathematical aspects. BMC Bioinforma. 17, S15. 10.1186/s12859-015-0857-9 PMC4959355 26821531

[B13] Bieluszewska A. Weglewska M. Bieluszewski T. Lesniewicz K. Poreba E. (2018). Pka-binding domain of akap 8 is essential for direct interaction with dpy 30 protein. FEBS J. 285, 947–964. 10.1111/febs.14378 29288530

[B14] Bose B. Bozdag S. (2019). “mirdriver: A tool to infer copy number derived mirna-gene networks in cancer,” in Proceedings of the 10th ACM international conference on bioinformatics, computational biology and health informatics, 366.

[B15] Bose B. Moravec M. Bozdag S. (2022). Computing microrna-gene interaction networks in pan-cancer using mirdriver. Sci. Rep. 12, 3717–17. 10.1038/s41598-022-07628-z 35260634 PMC8904490

[B16] Bueno D. F. Sunaga D. Y. Kobayashi G. S. Aguena M. Raposo-Amaral C. E. Masotti C. (2011). Human stem cell cultures from cleft lip/palate patients show enrichment of transcripts involved in extracellular matrix modeling by comparison to controls. Stem Cell. Rev. Rep. 7, 446–457. 10.1007/s12015-010-9197-3 21052871 PMC3073041

[B17] Cerhan J. R. Berndt S. I. Vijai J. Ghesquières H. McKay J. Wang S. S. (2014). Genome-wide association study identifies multiple susceptibility loci for diffuse large b cell lymphoma. Nat. Genet. 46, 1233–1238. 10.1038/ng.3105 25261932 PMC4213349

[B18] Chappell K. Manna K. Washam C. L. Graw S. Alkam D. Thompson M. D. (2021). Multi-omics data integration reveals correlated regulatory features of triple negative breast cancer. Mol. Omics 17, 677–691. 10.1039/d1mo00117e 34142686 PMC8504614

[B19] Consortium E. P. (2012). An integrated encyclopedia of dna elements in the human genome. Nature 489, 57–74. 10.1038/nature11247 22955616 PMC3439153

[B20] Consortium G. O. (2021). The gene ontology resource: Enriching a gold mine. Nucleic Acids Res. 49, D325–D334. 10.1093/nar/gkaa1113 33290552 PMC7779012

[B21] Corominas-Faja B. Cuyàs E. Gumuzio J. Bosch-Barrera J. Leis O. Martin Á. G. (2014). Chemical inhibition of acetyl-coa carboxylase suppresses self-renewal growth of cancer stem cells. Oncotarget 5, 8306–8316. 10.18632/oncotarget.2059 25246709 PMC4226684

[B22] Corrado C. Fontana S. (2020). Hypoxia and hif signaling: One axis with divergent effects. Int. J. Mol. Sci. 21, 5611. 10.3390/ijms21165611 32764403 PMC7460602

[B23] Csardi G. Nepusz T. (2006). The igraph software package for complex network research. Cambridge, MA: NECSI, 1695.

[B24] D’Aloia A. Berruti G. Costa B. Schiller C. Ambrosini R. Pastori V. (2018). Ralgps2 is involved in tunneling nanotubes formation in 5637 bladder cancer cells. Exp. Cell. Res. 362, 349–361. 10.1016/j.yexcr.2017.11.036 29208460

[B25] de Heer E. C. Jalving M. Harris A. L. (2020). Hifs, angiogenesis, and metabolism: Elusive enemies in breast cancer. J. Clin. investigation 130, 5074–5087. 10.1172/JCI137552 PMC7524491 32870818

[B26] De Tayrac M. Lê S. Aubry M. Mosser J. Husson F. (2009). Simultaneous analysis of distinct omics data sets with integration of biological knowledge: Multiple factor analysis approach. BMC genomics 10, 32. 10.1186/1471-2164-10-32 19154582 PMC2636827

[B27] Dorantes-Gilardi R. García-Cortés D. Hernández-Lemus E. Espinal-Enríquez J. (2021). k-core genes underpin structural features of breast cancer. Sci. Rep. 11, 16284–16317. 10.1038/s41598-021-95313-y 34381069 PMC8358063

[B28] Drago-García D. Espinal-Enríquez J. Hernández-Lemus E. (2017). Network analysis of emt and met micro-rna regulation in breast cancer. Sci. Rep. 7, 13534. 10.1038/s41598-017-13903-1 29051564 PMC5648819

[B29] Fan Z. Zhou Y. Ressom H. W. (2020). Mota: Network-based multi-omic data integration for biomarker discovery. Metabolites 10, 144. 10.3390/metabo10040144 32276350 PMC7241240

[B30] Farrugia M. Sharma S. Lin C. McLaughlin S. Vanderbilt D. Ammer A. (2015). Regulation of anti-apoptotic signaling by kruppel-like factors 4 and 5 mediates lapatinib resistance in breast cancer. Cell. death Dis. 6, e1699. 10.1038/cddis.2015.65 25789974 PMC4385942

[B31] Fernández-Centeno E. de Ojeda G. Rojo J. M. Portolés P. (2000). Crry/p65, a membrane complement regulatory protein, has costimulatory properties on mouse t cells. J. Immunol. 164, 4533–4542. 10.4049/jimmunol.164.9.4533 10779754

[B32] Fu N. Y. Rios A. C. Pal B. Soetanto R. Lun A. T. Liu K. (2015). Egf-mediated induction of mcl-1 at the switch to lactation is essential for alveolar cell survival. Nat. Cell. Biol. 17, 365–375. 10.1038/ncb3117 25730472

[B33] Furukawa-Hibi Y. Nagai T. Yun J. Yamada K. (2015). Stress increases dna methylation of the neuronal pas domain 4 (npas4) gene. Neuroreport 26, 827–832. 10.1097/WNR.0000000000000430 26222956

[B34] Garali I. Adanyeguh I. M. Ichou F. Perlbarg V. Seyer A. Colsch B. (2018). A strategy for multimodal data integration: Application to biomarkers identification in spinocerebellar ataxia. Briefings Bioinforma. 19, 1356–1369. 10.1093/bib/bbx060 29106465

[B35] García-Cortés D. de Anda-Jáuregui G. Fresno C. Hernández-Lemus E. Espinal-Enríquez J. (2020). Gene co-expression is distance-dependent in breast cancer. Front. Oncol. 10, 1232. 10.3389/fonc.2020.01232 32850369 PMC7396632

[B36] García-Cortés D. Hernández-Lemus E. Espinal-Enríquez J. (2021). Luminal a breast cancer co-expression network: Structural and functional alterations. Front. Genet. 12, 629475. 10.3389/fgene.2021.629475 33959148 PMC8096206

[B37] Gehlenborg N. (2019). UpSetR: A more scalable alternative to venn and euler diagrams for visualizing intersecting sets. Oxford, England: Oxford Academic.

[B38] Grand R. S. Burger L. Gräwe C. Michael A. K. Isbel L. Hess D. (2021). Banp opens chromatin and activates cpg-island-regulated genes. Nature 596, 133–137. 10.1038/s41586-021-03689-8 34234345

[B39] Gustavsen A., J. Pai S. Isserlin R. (2019). Rcy3: Network biology using cytoscape from within r. F1000Research 10.12688/f1000research.20887.3 PMC6880260 31819800

[B40] Han H. Shim H. Shin D. Shim J. E. Ko Y. Shin J. (2015). Trrust: A reference database of human transcriptional regulatory interactions. Sci. Rep. 5, 11432–11511. 10.1038/srep11432 26066708 PMC4464350

[B41] Hazelett C. C. Yeaman C. (2012). Sec5 and exo84 mediate distinct aspects of rala-dependent cell polarization. PLoS One 7, e39602. 10.1371/journal.pone.0039602 22761837 PMC3382198

[B42] He C. Imai M. Song H. Quigg R. J. Tomlinson S. (2005). Complement inhibitors targeted to the proximal tubule prevent injury in experimental nephrotic syndrome and demonstrate a key role for c5b-9. J. Immunol. 174, 5750–5757. 10.4049/jimmunol.174.9.5750 15843577

[B43] Hernández-de Diego R. Tarazona S. Martínez-Mira C. Balzano-Nogueira L. Furió-Tarí P. Pappas G. J. (2018). Paintomics 3: A web resource for the pathway analysis and visualization of multi-omics data. Nucleic acids Res. 46, W503-W509. 10.1093/nar/gky466 29800320 PMC6030972

[B44] Hollander D. Donyo M. Atias N. Mekahel K. Melamed Z. Yannai S. (2016). A network-based analysis of colon cancer splicing changes reveals a tumorigenesis-favoring regulatory pathway emanating from elk1. Genome Res. 26, 541–553. 10.1101/gr.193169.115 26860615 PMC4817777

[B45] Huang S. Chaudhary K. Garmire L. X. (2017). More is better: Recent progress in multi-omics data integration methods. Front. Genet. 8, 84. 10.3389/fgene.2017.00084 28670325 PMC5472696

[B46] Huang S. Xu W. Hu P. Lakowski T. M. (2019). Integrative analysis reveals subtype-specific regulatory determinants in triple negative breast cancer. Cancers 11, 507. 10.3390/cancers11040507 30974831 PMC6521146

[B47] Izquierdo A. G. Boughanem H. Diaz-Lagares A. Arranz-Salas I. Esteller M. Tinahones F. J. (2021). Dna methylome in visceral adipose tissue can discriminate patients with and without colorectal cancer. Epigenetics 1–12, 665–676. 10.1080/15592294.2021.1950991 PMC9235880 34311674

[B48] Jafarzadeh M. Soltani B. M. Soleimani M. Hosseinkhani S. (2020). Epigenetically silenced linc02381 functions as a tumor suppressor by regulating pi3k-akt signaling pathway. Biochimie 171, 63–71. 10.1016/j.biochi.2020.02.009 32092325

[B49] Jiang C. Xuan Z. Zhao F. Zhang M. Q. (2007). Tred: A transcriptional regulatory element database, new entries and other development. Nucleic acids Res. 35, D137–D140. 10.1093/nar/gkl1041 17202159 PMC1899102

[B50] Kanehisa M. Goto S. (2000). Kegg: Kyoto encyclopedia of genes and genomes. Nucleic acids Res. 28, 27–30. 10.1093/nar/28.1.27 10592173 PMC102409

[B51] Ke H. Wu Y. Wang R. Wu X. (2020). Creation of a prognostic risk prediction model for lung adenocarcinoma based on gene expression, methylation, and clinical characteristics. Med. Sci. Monit. Int. Med. J. Exp. Clin. Res. 26, 9258333–e925841. 10.12659/MSM.925833 PMC7549534 33021972

[B52] Kristensen V. N. Lingjærde O. C. Russnes H. G. Vollan H. K. M. Frigessi A. Børresen-Dale A.-L. (2014). Principles and methods of integrative genomic analyses in cancer. Nat. Rev. Cancer 14, 299–313. 10.1038/nrc3721 24759209

[B53] Kuijjer M. L. Fagny M. Marin A. Quackenbush J. Glass K. (2020). Puma: Panda using microrna associations. Bioinformatics 36, 4765–4773. 10.1093/bioinformatics/btaa571 32860050 PMC7750953

[B54] Lambert S. A. Jolma A. Campitelli L. F. Das P. K. Yin Y. Albu M. (2018). The human transcription factors. Cell. 172, 650–665. 10.1016/j.cell.2018.01.029 29425488 PMC12908702

[B55] Li J. Xu Y.-H. Lu Y. Ma X.-P. Chen P. Luo S.-W. (2013). Identifying differentially expressed genes and small molecule drugs for prostate cancer by a bioinformatics strategy. Asian Pac. J. cancer Prev. 14, 5281–5286. 10.7314/apjcp.2013.14.9.5281 24175814

[B56] Li W. Zhang S. Liu C.-C. Zhou X. J. (2012). Identifying multi-layer gene regulatory modules from multi-dimensional genomic data. Bioinformatics 28, 2458–2466. 10.1093/bioinformatics/bts476 22863767 PMC3463121

[B57] Li Y. Wang Y. Wen Y. Zhang T. Wang X. Jiang C. (2022). Whole-exome sequencing of a cohort of infertile men reveals novel causative genes in teratozoospermia that are chiefly related to sperm head defects. Hum. Reprod. 37, 152–177. 10.1093/humrep/deab229 34791246

[B58] Li Y. Zhao X. Liu Q. Liu Y. (2021). Bioinformatics reveal macrophages marker genes signature in breast cancer to predict prognosis. Ann. Med. 53, 1019–1031. 10.1080/07853890.2021.1914343 34187256 PMC8253219

[B59] Liang Y.-K. Lin H.-Y. Dou X.-W. Chen M. Wei X.-L. Zhang Y.-Q. (2018). Mir-221/222 promote epithelial-mesenchymal transition by targeting notch3 in breast cancer cell lines. NPJ breast cancer 4, 20–29. 10.1038/s41523-018-0073-7 30109262 PMC6079079

[B60] Liu J. Liang G. Siegmund K. D. Lewinger J. P. (2018). Data integration by multi-tuning parameter elastic net regression. BMC Bioinforma. 19, 369. 10.1186/s12859-018-2401-1 PMC6180486 30305021

[B61] Liu J. Liu X. Zhou G. Xiao R. Cao Y. (2012). Conditioned medium from chondrocyte/scaffold constructs induced chondrogenic differentiation of bone marrow stromal cells. Anatomical Rec. Adv. Integr. Anat. Evol. Biol. 295, 1109–1116. 10.1002/ar.22500 22644958

[B62] Luo Y. Sun F. Peng X. Dong D. Ou W. Xie Y. (2021). Integrated bioinformatics analysis to identify abnormal methylated differentially expressed genes for predicting prognosis of human colon cancer. Int. J. General Med. 14, 4745–4756. 10.2147/IJGM.S324483 PMC8403012 34466019

[B63] Maksimovic J. Gagnon-Bartsch J. A. Speed T. P. Oshlack A. (2015). Removing unwanted variation in a differential methylation analysis of illumina humanmethylation450 array data. Nucleic acids Res. 43, e106. 10.1093/nar/gkv526 25990733 PMC4652745

[B64] Marbach D. Lamparter D. Quon G. Kellis M. Kutalik Z. Bergmann S. (2016). Tissue-specific regulatory circuits reveal variable modular perturbations across complex diseases. Nat. methods 13, 366–370. 10.1038/nmeth.3799 26950747 PMC4967716

[B65] Margolin A. A. Nemenman I. Basso K. Wiggins C. Stolovitzky G. Dalla Favera R. (2006). Aracne: An algorithm for the reconstruction of gene regulatory networks in a mammalian cellular context. BMC Bioinforma. Biomed. Cent. 7, S7. 10.1186/1471-2105-7-S1-S7 PMC1810318 16723010

[B66] Matic I. van Hagen M. Schimmel J. Macek B. Ogg S. C. Tatham M. H. (2008). *In vivo* identification of human small ubiquitin-like modifier polymerization sites by high accuracy mass spectrometry and an *in vitro* to *in vivo* strategy. Mol. Cell. proteomics 7, 132–144. 10.1074/mcp.M700173-MCP200 17938407 PMC3840926

[B67] Meng C. Basunia A. Peters B. Gholami A. M. Kuster B. Culhane A. C. (2019). Mogsa: Integrative single sample gene-set analysis of multiple omics data. Mol. Cell. Proteomics 18, S153-S168–S168. 10.1074/mcp.TIR118.001251 31243065 PMC6692785

[B68] Messaoudene M. Mourikis T. Michels J. Fu Y. Bonvalet M. Lacroix-Trikki M. (2019). T-Cell bispecific antibodies in node-positive breast cancer: Novel therapeutic avenue for mhc class i loss variants. Ann. Oncol. 30, 934–944. 10.1093/annonc/mdz112 30924846 PMC7614969

[B69] Meyer P. E. (2014). Infotheo: Information-Theoretic measures. Princeton, NJ: R. package.

[B70] Moldogazieva N. T. Mokhosoev I. M. Terentiev A. A. (2020). Metabolic heterogeneity of cancer cells: An interplay between hif-1, gluts, and ampk. Cancers 12, 862. 10.3390/cancers12040862 32252351 PMC7226606

[B71] Mukherjee S. Asnani H. Kannan S. (2020). “Ccmi: Classifier based conditional mutual information estimation,” in Proceedings of Machine Learning Research.

[B72] Nakazawa T. Nakano I. Sato M. Nakamura T. Tamai M. Mori N. (2002). Comparative expression profiles of trk receptors and shc-related phosphotyrosine adapters during retinal development: Potential roles of n-shc/shcc in brain-derived neurotrophic factor signal transduction and modulation. J. Neurosci. Res. 68, 668–680. 10.1002/jnr.10259 12111828

[B73] Neph S. Stergachis A. B. Reynolds A. Sandstrom R. Borenstein E. Stamatoyannopoulos J. A. (2012). Circuitry and dynamics of human transcription factor regulatory networks. Cell. 150, 1274–1286. 10.1016/j.cell.2012.04.040 22959076 PMC3679407

[B74] Network C. G. A. (2012). Comprehensive molecular portraits of human breast tumours. Nature 490, 61–70. 10.1038/nature11412 23000897 PMC3465532

[B75] Nueda M. J. Ferrer A. Conesa A. (2012). Arsyn: A method for the identification and removal of systematic noise in multifactorial time course microarray experiments. Biostatistics 13, 553–566. 10.1093/biostatistics/kxr042 22085896

[B76] Ochoa S. de Anda-Jáuregui G. Hernández-Lemus E. (2021). An information theoretical multilayer network approach to breast cancer transcriptional regulation. Front. Genet. 12, 617512. 10.3389/fgene.2021.617512 33815463 PMC8014033

[B77] Pathak B. R. Breed A. A. Apte S. Acharya K. Mahale S. D. (2016). Cysteine-rich secretory protein 3 plays a role in prostate cancer cell invasion and affects expression of psa and anxa1. Mol. Cell. Biochem. 411, 11–21. 10.1007/s11010-015-2564-2 26369530

[B78] Patra S. K. (2008). Ras regulation of dna-methylation and cancer. Exp. Cell. Res. 314, 1193–1201. 10.1016/j.yexcr.2008.01.012 18282569

[B79] Pethel S. D. Hahs D. W. (2014). Exact test of independence using mutual information. Entropy 16, 2839–2849. 10.3390/e16052839

[B80] Piao H.-l. Yuan Y. Wang M. Sun Y. Liang H. Ma L. (2014). *α*-catenin acts as a tumour suppressor in e-cadherin-negative basal-like breast cancer by inhibiting nf-*κ*b signalling. Nat. Cell. Biol. 16, 245–254. 10.1038/ncb2909 24509793 PMC3943677

[B81] Privat M. Rudewicz J. Sonnier N. Tamisier C. Ponelle-Chachuat F. Bignon Y.-J. (2018). Antioxydation and cell migration genes are identified as potential therapeutic targets in basal-like and brca1 mutated breast cancer cell lines. Int. J. Med. Sci. 15, 46–58. 10.7150/ijms.20508 29333087 PMC5765739

[B82] Pupa S. M. Ligorio F. Cancila V. Franceschini A. Tripodo C. Vernieri C. (2021). Her2 signaling and breast cancer stem cells: The bridge behind her2-positive breast cancer aggressiveness and therapy refractoriness. Cancers 13, 4778. 10.3390/cancers13194778 34638263 PMC8507865

[B83] R Core Team (2021). R: A language and environment for statistical computing. Vienna, Austria: R Foundation for Statistical Computing.

[B84] Ramdzan Z. M. Vadnais C. Pal R. Vandal G. Cadieux C. Leduy L. (2014). Ras transformation requires cux1-dependent repair of oxidative dna damage. PLoS Biol. 12, e1001807. 10.1371/journal.pbio.1001807 24618719 PMC3949673

[B85] Risso D. Schwartz K. Sherlock G. Dudoit S. (2011). GC-content normalization for RNA-seq data. BMC Bioinforma. 12, 480. 10.1186/1471-2105-12-480 PMC3315510 22177264

[B86] Rohart F. Gautier B. Singh A. Le Cao K.-A. (2017). mixomics: An r package for ‘omics feature selection and multiple data integration. PLoS Comput. Biol. 13, e1005752. 10.1371/journal.pcbi.1005752 29099853 PMC5687754

[B87] Rose N. R. Klose R. J. (2014). Understanding the relationship between dna methylation and histone lysine methylation. Biochimica Biophysica Acta (BBA)-Gene Regul. Mech. 1839, 1362–1372. 10.1016/j.bbagrm.2014.02.007 PMC4316174 24560929

[B88] Ru Y. Kechris K. J. Tabakoff B. Hoffman P. Radcliffe R. A. Bowler R. (2014). The multimir r package and database: Integration of microrna–target interactions along with their disease and drug associations. Nucleic acids Res. 42, e133. 10.1093/nar/gku631 25063298 PMC4176155

[B89] Schulz D. M. Bollner C. Thomas G. Atkinson M. Esposito I. Hofler H. (2009). Identification of differentially expressed proteins in triple-negative breast carcinomas using dige and mass spectrometry. J. proteome Res. 8, 3430–3438. 10.1021/pr900071h 19485423

[B90] Shamay M. Greenway M. Liao G. Ambinder R. F. Hayward S. D. (2010). De novo dna methyltransferase dnmt3b interacts with nedd8-modified proteins. J. Biol. Chem. 285, 36377–36386. 10.1074/jbc.M110.155721 20847044 PMC2978566

[B91] Shannon P. Markiel A. Ozier O. Baliga N. S. Wang J. T. Ramage D. (2003). Cytoscape: A software environment for integrated models of biomolecular interaction networks. Genome Res. 13, 2498–2504. 10.1101/gr.1239303 14597658 PMC403769

[B92] Sohn K.-A. Kim D. Lim J. Kim J. H. (2013). Relative impact of multi-layered genomic data on gene expression phenotypes in serous ovarian tumors. BMC Syst. Biol. 7, S9. 10.1186/1752-0509-7-S6-S9 PMC3906601 24521303

[B93] Sonawane A. R. DeMeo D. L. Quackenbush J. Glass K. (2021). Constructing gene regulatory networks using epigenetic data. npj Syst. Biol. Appl. 7, 45–13. 10.1038/s41540-021-00208-3 34887443 PMC8660777

[B94] Strelnikov V. V. Kuznetsova E. B. Tanas A. S. Rudenko V. V. Kalinkin A. I. Poddubskaya E. V. (2021). Abnormal promoter dna hypermethylation of the integrin, nidogen, and dystroglycan genes in breast cancer. Sci. Rep. 11, 2264–2314. 10.1038/s41598-021-81851-y 33500458 PMC7838398

[B95] Sugiyama A. Okada M. Yamawaki H. (2020). Canstatin suppresses isoproterenol-induced cardiac hypertrophy through inhibition of calcineurin/nuclear factor of activated t-cells pathway in rats. Eur. J. Pharmacol. 871, 172849. 10.1016/j.ejphar.2019.172849 31843516

[B96] Sumi E. Iehara N. Akiyama H. Matsubara T. Mima A. Kanamori H. (2007). Sry-related hmg box 9 regulates the expression of col4a2 through transactivating its enhancer element in mesangial cells. Am. J. pathology 170, 1854–1864. 10.2353/ajpath.2007.060899 PMC1899455 17525254

[B97] Tam S. Tsao M.-S. McPherson J. D. (2015). Optimization of mirna-seq data preprocessing. Briefings Bioinforma. 16, 950–963. 10.1093/bib/bbv019 PMC4652620 25888698

[B98] Tan Z. Niu B. Tsang K. Y. Melhado I. G. Ohba S. He X. (2018). Synergistic co-regulation and competition by a sox9-gli-foxa phasic transcriptional network coordinate chondrocyte differentiation transitions. PLoS Genet. 14, e1007346. 10.1371/journal.pgen.1007346 29659575 PMC5919691

[B99] Tapia-Carrillo D. Tovar H. Velazquez-Caldelas T. E. Hernandez-Lemus E. (2019). Master regulators of signaling pathways: An application to the analysis of gene regulation in breast cancer. Front. Genet. 10, 1180. 10.3389/fgene.2019.01180 31850059 PMC6902642

[B100] Tarazona S. Furió-Tarí P. Turrà D. Pietro A. D. Nueda M. J. Ferrer A. (2015). Data quality aware analysis of differential expression in rna-seq with noiseq r/bioc package. Nucleic acids Res. 43, e140. 10.1093/nar/gkv711 26184878 PMC4666377

[B101] Tenenhaus A. Philippe C. Guillemot V. Le Cao K.-A. Grill J. Frouin V. (2014). Variable selection for generalized canonical correlation analysis. Biostatistics 15, 569–583. 10.1093/biostatistics/kxu001 24550197

[B102] Thammaiah C. K. Jayaram S. (2016). Role of let-7 family microrna in breast cancer. Non-coding RNA Res. 1, 77–82. 10.1016/j.ncrna.2016.10.003 PMC6096426 30159414

[B103] Vidal Ocabo E. Sayols S. Moran S. Guillaumet-Adkins A. Schroeder M. P. Royo R. (2017). A dna methylation map of human cancer at single base-pair resolution. Oncogene 36 (40), 5648–5657. 10.1038/onc.2017.176 28581523 PMC5633654

[B104] Weigel Muñoz M. Carvajal G. Curci L. Gonzalez S. N. Cuasnicu P. S. (2019). Relevance of crisp proteins for epididymal physiology, fertilization, and fertility. Andrology 7, 610–617. 10.1111/andr.12638 31218833

[B105] Weighill D. Burkholz R. Guebila M. B. Zacharias H. U. Quackenbush J. Altenbuchinger M. (2021). DRAGON: Determining regulatory associations using graphical models on multi-omic networks. Oxford, England: Nucleic Acids Res. [Epub ahead of print]. 10.1093/nar/gkac1157 PMC9943674 36533448

[B106] Wickham H. (2016). ggplot2: Elegant graphics for data analysis. New York: Springer-Verlag.

[B107] Wollen E. J. Sejersted Y. Wright M. S. Bik-Multanowski M. Madetko-Talowska A. Günther C.-C. (2013). Transcriptome profiling of the newborn mouse lung after hypoxia and reoxygenation: Hyperoxic reoxygenation affects mtor signaling pathway, dna repair, and jnk-pathway regulation. Pediatr. Res. 74, 536–544. 10.1038/pr.2013.140 23999071

[B108] Wright K. L. Adams J. R. Liu J. C. Loch A. J. Wong R. G. Jo C. E. (2015). Ras signaling is a key determinant for metastatic dissemination and poor survival of luminal breast cancer patients. Cancer Res. 75, 4960–4972. 10.1158/0008-5472.CAN-14-2992 26400062

[B109] Wu J. Wang C. Miao X. Wu Y. Yuan J. Ding M. (2017). Age-related insulin-like growth factor binding protein-4 overexpression inhibits osteogenic differentiation of rat mesenchymal stem cells. Cell. Physiology Biochem. 42, 640–650. 10.1159/000477873 28595186

[B110] Wu T. Hu E. Xu S. Chen M. Guo P. Dai Z. (2021). Clusterprofiler 4.0: A universal enrichment tool for interpreting omics data. Innovation 2, 100141. 10.1016/j.xinn.2021.100141 34557778 PMC8454663

[B111] Xin S. Fang W. Li J. Li D. Wang C. Huang Q. (2021). Impact of stat1 polymorphisms on crizotinib-induced hepatotoxicity in alk-positive non-small cell lung cancer patients. J. Cancer Res. Clin. Oncol. 147, 725–737. 10.1007/s00432-020-03476-4 33387041 PMC11801888

[B112] Xiong B. Lei X. Zhang L. Fu J. (2017). mir-103 regulates triple negative breast cancer cells migration and invasion through targeting olfactomedin 4. Biomed. Pharmacother. 89, 1401–1408. 10.1016/j.biopha.2017.02.028 28320108

[B113] Xu N. Wu Y.-P. Ke Z.-B. Liang Y.-C. Cai H. Su W.-T. (2019). Identification of key dna methylation-driven genes in prostate adenocarcinoma: An integrative analysis of tcga methylation data. J. Transl. Med. 17, 311–315. 10.1186/s12967-019-2065-2 31533842 PMC6751626

[B114] Xu X. Zhang M. Xu F. Jiang S. (2020). Wnt signaling in breast cancer: Biological mechanisms, challenges and opportunities. Mol. cancer 19, 165–235. 10.1186/s12943-020-01276-5 33234169 PMC7686704

[B115] Zamora-Fuentes J. M. Hernández-Lemus E. Espinal-Enríquez J. (2022). Oncogenic role of mir-217 during clear cell renal carcinoma progression. Front. Oncol. 12, 934711. 10.3389/fonc.2022.934711 35936681 PMC9354686

[B116] Zhang J. Huang K. (2017). Pan-cancer analysis of frequent dna co-methylation patterns reveals consistent epigenetic landscape changes in multiple cancers. Bmc Genomics 18, 1045–1114. 10.1186/s12864-016-3259-0 28198667 PMC5310283

[B117] Zhang P. Wen X. Gu F. Deng X. Li J. Dong J. (2013). Methylation profiling of serum dna from hepatocellular carcinoma patients using an infinium human methylation 450 beadchip. Hepatol. Int. 7, 893–900. 10.1007/s12072-013-9437-0 26201927

[B118] Zhao S. Geybels M. S. Leonardson A. Rubicz R. Kolb S. Yan Q. (2017). Epigenome-Wide tumor DNA methylation profiling identifies novel prognostic biomarkers of metastatic-lethal progression in men diagnosed with clinically localized prostate cancer. Clin. Cancer Res. 23, 311–319. 10.1158/1078-0432.CCR-16-0549 27358489 PMC5199634

[B119] Zheng G. Tu K. Yang Q. Xiong Y. Wei C. Xie L. (2008). Itfp: An integrated platform of mammalian transcription factors. Bioinformatics 24, 2416–2417. 10.1093/bioinformatics/btn439 18713790

