# Supplementary material for: Functional impact of multi-omic interactions in breast cancer subtypes
Source: Front Genet. 2023 Jan 5;13:1078609. doi: 10.3389/fgene.2022.1078609 (PMC9850112; doi:10.3389/fgene.2022.1078609)
Supplement: Supplementary file 3 [file DataSheet1.PDF]

Supplementary Material

1 SUPPLEMENTARY DATA

2 SUPPLEMENTARY TABLES AND FIGURES

2.1 Tables

Table S1. Functional enrichment results for the gene ontology of biological processes.

Table S2. Functional enrichment results for the KEGG pathways

Table S3. Clustering results for functions enriched in the four breast cancer subtypes and the normal tissue

2.2 Figures

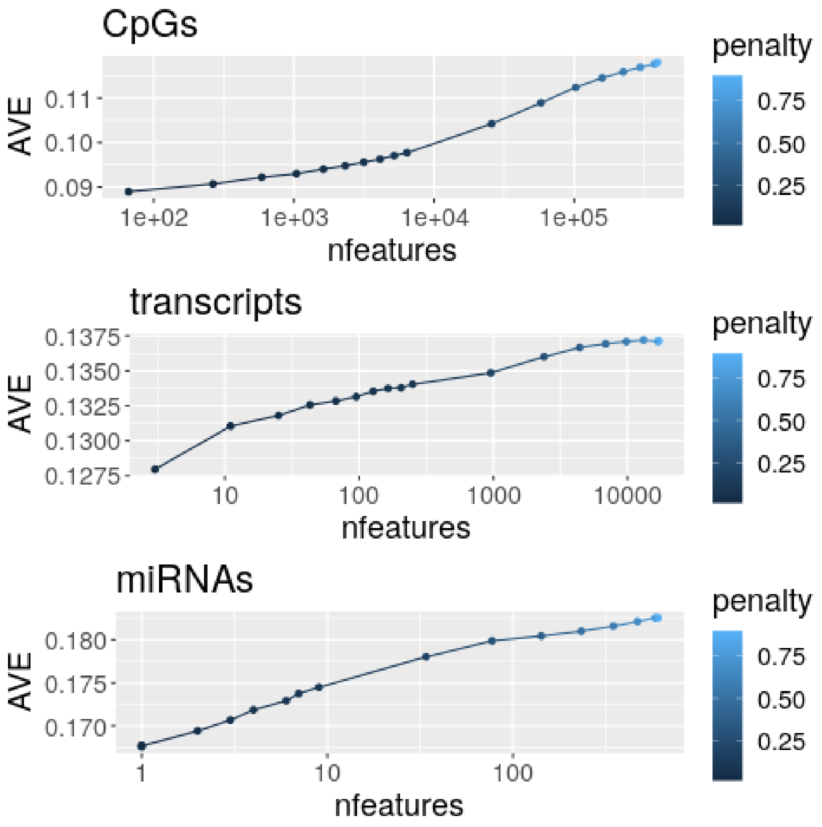

Figure S1. Selection of the sparsity parameters. Sparsity parameters were fitted by cross-validation, testing 18 values within [0.01,...,0.09,0.1,..,0.9]. Each combination of three values, for the three omics, was tested 10 times, adding a total of 11340 instances for every value per omic. The median number of features (nfeatures) and average variance explained (AVE), no matter the other two omics, is shown as a dot. We choose the dot before the largest change in slope, namely, 0.02 for the CpG sites and transcripts, and 0.05 for the microRNAs.

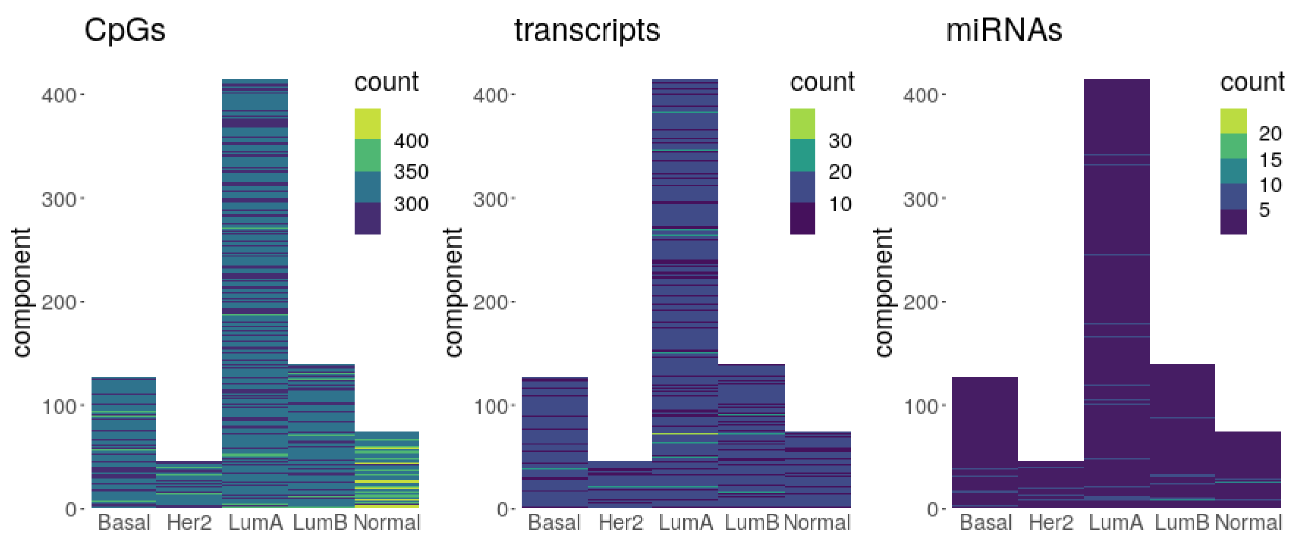

Figure S2. Selected features per subtype. Each row is a different matrix component and the color indicates how many features were selected in the component

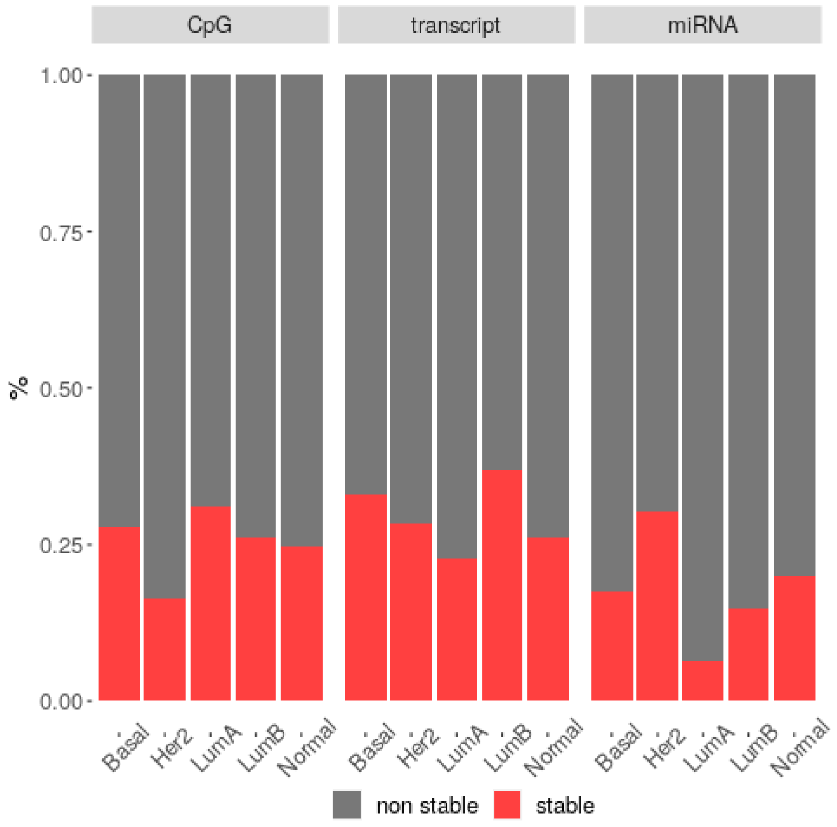

Figure S3. Proportion of stable features per omic and dataset

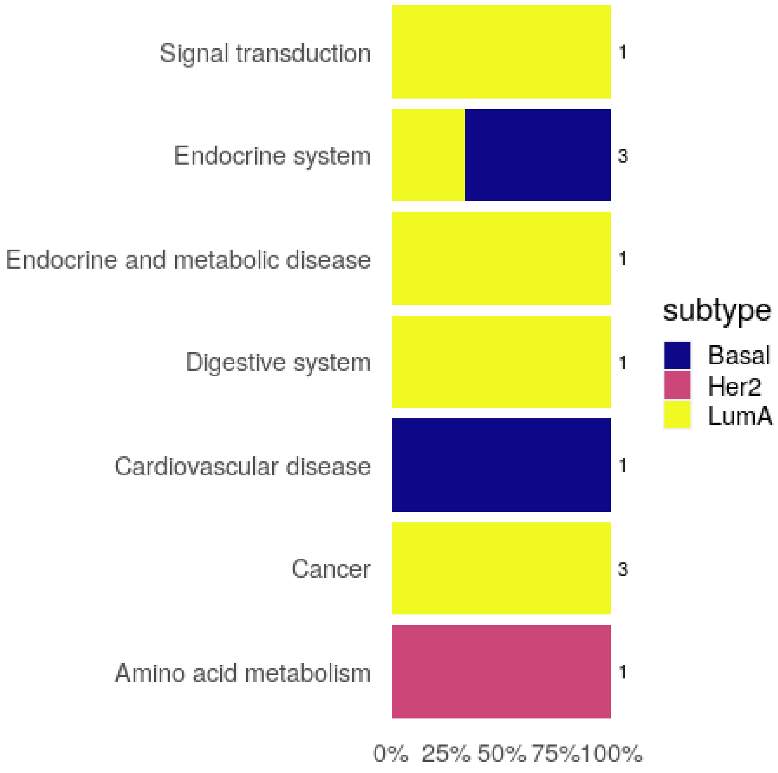

Figure S4. Exclusive KEGG classes. There is no significant bias for any of these categories
